# Asymmetrical color filling-in from the nasal to the temporal side of the blind spot

**DOI:** 10.3389/fnhum.2014.00534

**Published:** 2014-07-18

**Authors:** Hui Li, Junxiang Luo, Yiliang Lu, Janis Kan, Lothar Spillmann, Wei Wang

**Affiliations:** State Key Laboratory of Neuroscience and Key Laboratory of Primate Neurobiology, Institute of Neuroscience, Shanghai Institutes for Biological Sciences, Chinese Academy of SciencesShanghai, China

**Keywords:** blind spot, color filling-in, retinotopic, eccentricity, cortical magnification factor

## Abstract

The physiological blind spot, corresponding to the optic disk in the retina, is a relatively large (6 × 8°) area in the visual field that receives no retinal input. However, we rarely notice the existence of it in daily life. This is because the blind spot fills in with the brightness, color, texture, and motion of the surround. The study of filling-in enables us to better understand the creative nature of the visual system, which generates perceptual information where there is none. Is there any retinotopic rule in the color filling-in of the blind spot? To find out, we used mono-colored and bi-colored annuli hugging the boundary of the blind spot. We found that mono-colored annuli filled in the blind spot uniformly. By contrast, bi-colored annuli, where one half had a given color, while the other half had a different one, filled in the blind spot asymmetrically. Specifically, the color surrounding the nasal half typically filled in about 75% of the blind spot area, whereas the color surrounding the temporal half filled in only about 25%. This asymmetry was dependent on the relative size of the half rings, but not the two colors used, and was absent when the bi-colored annulus was rotated by 90°. Here, the two colors on the upper and lower sides of the blind spot filled in the enclosed area equally. These results suggest that the strength of filling-in decreases with distance from the fovea consistent with the decrease of the cortical magnification factor.

## Introduction

The optic disk of the eye is formed by the ganglion cell axons exiting the eyeball on their way to the brain, resulting in a region on the retina where there are no photoreceptors and thus there should be no vision at all. This area is known as the physiological blind spot which is located in the temporal visual field with its center at approximately 15° and subtending 6 × 8° of visual angle (Armaly, 1969; Ramachandran, 1992b; Pessoa and De Weerd, 2003; Komatsu, 2006). Although the blind spot has been revealed by retinotopic mapping of the human primary visual cortex (V1) (Tootell et al., 1998; Awater et al., 2005), we never experience a dark hole in our visual field; instead, we perceive a complete visual world. This has two reasons: first, the region of the visual field corresponding to the blind spot of one eye is covered by the fellow eye, and in this way the left and right eyes compensate for each other’s blind spots in binocular vision. It was also found electrophysiologically that most V1 neurons inside and outside of the cortical representation of the optic disk are binocularly driven (Fiorani et al., 1992; Komatsu et al., 2000). Second, we rarely notice the existence of the blind spot even in monocular vision. This is because the blind spot acquires the brightness, color, texture and even motion from the surround, a phenomenon known as perceptual filling-in (Walls, 1954; Gerrits and Vendrik, 1970; Ramachandran, 1992a) and attributable to spreading of information from the edge by way of lateral propagation (Spillmann, 2011). Additionally, Komatsu et al. (2000) have shown that although there is no direct retinal input to the cortical region corresponding to the blind spot, the blind spot region may become filled in by the intracortical circuitry, resulting in large receptive fields capable of interpolating retinal signals in the interest of contiguous surface and contour perception (Komatsu, 2011).

Filling-in has long been assumed to proceed from the surround inward to the center of a target (Troxler, 1804; Krauskopf, 1963; Spillmann et al., 1984b; Paradiso and Hahn, 1996), resulting in uniform filling-in with the surround color (Ramachandran, 1992a; Brown and Thurmond, 1993). Even a thin colored border surrounding the blind spot suffices to generate the perception of a uniformly colored surface filling in the blind spot (Spillmann et al., 2006). However, when an artificial scotoma was used consisting of a central disk surrounded by one or two annuli, color filling-in was found to be bi-directional, with the color spreading either inward towards the center or outward from the center (Hamburger et al., 2006). Color filling-in or filling-out was shown to correlate with the diameter of the target (Shimojo et al., 2003; Kanai et al., 2006). Furthermore, the time for perceptual completion of an artificial scotoma was found to correlate directly with the size of the cortical projection by the artificial scotoma, but not necessarily the eccentricity of the scotoma *per se* (De Weerd et al., 1998). Other studies have shown that the time required for filling-in decreases with increasing retinal eccentricity of the target (Ramachandran et al., 1993; De Weerd et al., 1998; Sakaguchi, 2001; Welchman and Harris, 2001; Proudlock et al., 2006). These observations in filling-in of an artificial scotoma suggest that the cortical magnification factor may play an important role.

Since the density of retinal photoreceptors decreases with increasing eccentricity (Osterberg, 1935; Curcio et al., 1987, 1990), it produces less cortical representation for more peripheral stimuli, resulting in a progressively smaller cortical magnification factor (Daniel and Whitteridge, 1961; Cowey and Rolls, 1974; Drasdo, 1977; Wandell and Winawer, 2011). In contrast to artificial scotomata (see Pessoa and De Weerd, 2003; Komatsu, 2006; Anstis, 2010 for reviews), the blind spot is part of the normal development of the visual system, resulting in filling-in that is not only rapid, but also pre-attentive. Here we specifically ask: Is there any retinotopic rule governing the color filling-in of the blind spot? For example, will the color on the proximal side of the blind spot fill in more extensively than the color on the distal side because of the larger magnification factor?

Most previous works studying the filling-in of the blind spot used stimuli that were uniform all around the blind spot (Ramachandran, 1992a; Brown and Thurmond, 1993; Durgin et al., 1995; Murakami, 1995; Spillmann et al., 2006). In a preliminary study, using a bi-colored ring, we observed strongly asymmetrical color filling-in of the blind spot proceeding from the nasal to the temporal half. All subjects reported that the filled-in color from the nasal side of the blind spot occupied a much larger region than the filled-in color from the temporal side.

To elucidate this asymmetry, we used mono-colored and bi-colored annuli hugging and slightly overlapping with the boundary of the blind spot.

## Materials and methods

### Subjects

Twelve subjects, 8 males and 4 females, all students from the Institute of Neuroscience, Shanghai Institutes for Biological Sciences, Chinese Academy of Sciences, and aged 24–30 years participated in the experiment. They had given written consent to the procedure in accordance with institutional guidelines and the Declaration of Helsinki. All had normal or corrected-to-normal vision and had no history of psychiatric or neurological disorders. Subjects were paid for their participation. Experiments were approved by the Ethics Committee of the Institute of Neuroscience, Shanghai Institutes of Biological Sciences, Chinese Academy of Sciences. Subjects were naïve to the project except for one, who is one of the authors, and all subjects practiced for approximately six trial sessions before data acquisition. Subjects sat in a dark room at a distance of 0.4 m from a HP P1230 CRT monitor that had a refresh rate of 85 Hz. At this distance, the screen of the monitor subtended a visual angle of 53.2°, within which the stimuli were presented. A chin-forehead rest was used to stabilize the head. Stimulus generation, presentation, and data acquisition were controlled by a personal computer running Matlab.

### Identification of the blind spot

The blind spot of each individual subject was mapped, using a customized computer-controlled procedure. Subject fixated on a white fixation point (68.5 cd/m^2^) presented on a dark background (0.10 cd/m^2^) with their right eye, while their untested, left eye was covered. A computer mouse controlled a small white test probe (0.67°, 34.3 cd/m^2^), which was slowly moved across the monitor screen by the experimenter to precisely map the blind spot. The positions where the probe disappeared and reappeared were digitally marked by clicking the mouse at those locations. The edge of the blind spot was measured twice for each condition to confirm the coordinates within the visual field. This process was repeated from 12 different directions, according to the numbers on the face of a clock. Thresholds for disappearance and reappearance were averaged for each meridian. The average size of the blind spot among our subjects was found to be 7.6° in width (corresponding to eccentricities of 13.6° and 21.2°) and 8.3° in height (2.8° to −5.5°) in the temporal half of the visual field, comparable with previous studies (Ramachandran, 1992b; Spillmann et al., 2006; Abadi et al., 2011).

### Visual stimuli and experimental procedure

All stimuli used in this study were generated in MATLAB software (MathWorks) using Psychtoolbox (Brainard, 1997; Pelli, 1997). Stimuli consisted of computer-generated mono-colored and bi-colored annuli, surrounding the blind spot boundary of each individual subject. In a recent study a ring as thin as 0.5° had been found to be sufficient for inducing color filling-in of the blind spot (Spillmann et al., 2006). The width of the mono- and bi-colored annuli in our study was therefore set at 2.5°. As there were individual differences in the size of the blind spot, the diameter of the stimuli was adjusted for each subject so that the annuli overlapped with the edge of the blind spot. The average size of the blind spot is slightly oval in our subjects. Therefore, the actual inner annulus we used was slightly elongated (elliptical), with the edge of the blind spot covered by the inner rim of the annuli.

**Figure 1:**
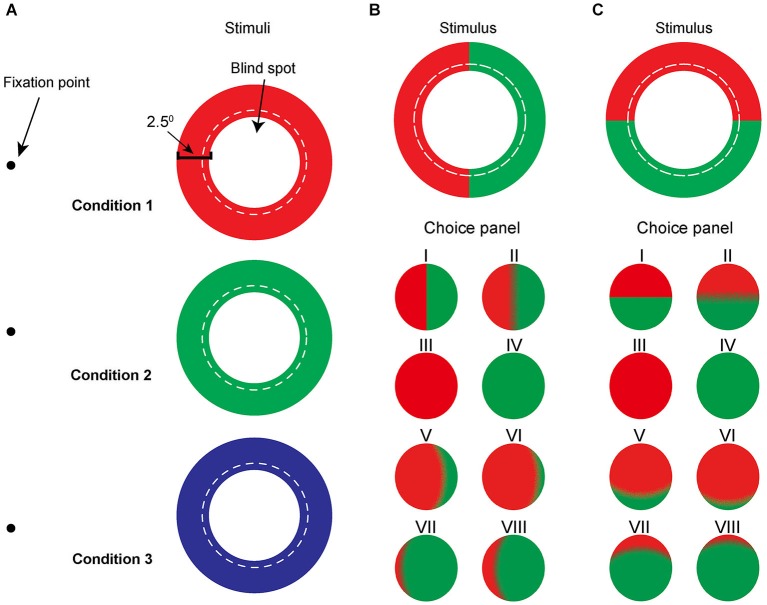
**Schematic illustration of the mono-colored annuli (A) and bi-colored annuli (B and C). (A)** Red, green, and blue mono-colored rings adjusted to hug the blind spot boundary were presented randomly on the screen while subjects fixated at a small fixation point. The width of the ring subtends 2.5° of the visual field. The dotted line represents the previously measured boundary of the blind spot. For the simplicity, we are using concentric circles rather than ellipses to depict the boundary of the blind spot. **(B)**, **(C)** Bi-colored annuli of the same size were subdivided either **(B)** vertically, i.e. nasal/temporally or **(C)** horizontally, i.e. upper/lower, using, for example, red and green. Bottom panel (category labeled choice panel) is an example of the eight cartoons representing different levels of filling-in for subjects to choose from.

The gamma of the monitor was carefully corrected by using the Color Calibration device (ColorCAL) from Cambridge VS system. Color filling-in was first tested using annuli with one color only (Figure 1A). This color was randomly chosen from red, green, and blue. CIE coordinates were as follows: red (*x* = 0.605, *y* = 0.333), blue (*x* = 0.152, *y* = 0.075), and green (*x* = 0.275, *y* = 0.591). Bi-colored rings where one half had one color and the other half a different color were generated using a random combination of any two of these three colors (Figures 1B,C). In one condition, the two halves were juxtaposed symmetrically on the nasal and temporal sides of the blind spot; and in the other, on the upper and lower sides of the blind spot. There were a total of 15 stimuli: 3 mono-colored stimuli and 12 bi-colored stimuli, 6 of which were nasal-temporal and the other 6 upper-lower configurations. All stimuli had a luminance of 12 cd/m^2^ measured by ColorCAl. Preliminary data indicated that our subjects found it easier to describe their perception when stimuli were presented on a white background rather than a black background, thus stimuli were all presented against a white (68.5 cd/m^2^) background.

The task was to report the perceptual filling-in of the blind spot by selecting a colored disk from a choice panel (Figure 1) that best resembled their observations. Subjects were asked to press a key on the keyboard to start a stimulus presentation, and to press the key again as soon as filling-in occurred (reaction time). Immediately after the second key press, the stimulus disappeared and a choice panel was presented on the same screen. Measured mean stimulus duration of bi-colored annuli was 1.80 ± 0.97 s (*n* = 12 subjects), which was the time it took for filling-in including the reaction time. Subjects repeated an observation until they were satisfied with their match. In the mono-colored trials, a panel of three computer-generated representations was shown to help subjects classify their observations: complete filling-in of the blind spot area, incomplete filling-in of the center of the annulus, and stimulus disappearance. By comparison, in the bi-colored condition, a 2-row panel of eight computer-generated representations of possible percepts was presented on the screen (Figure 1, Choice panels I–VIII). Each choice disk size was 7.5° in diameter and separated by 4° from its neighbors. All choices in mono-colored and bi-colored trials were based on drawings previously made by subjects in a preliminary experiment, where stimuli were cut from poster paper and subjects were asked to describe as well as draw their observations. Choices V and VI were constructed to describe asymmetrical color filling-in from the nasal (or upper) to the temporal (or lower) sides of the blind spot, whereas choices VII and VIII were used to describe the asymmetry in the opposite direction. These disks were presented to give subjects a more complete range of choices.

In a set of complementary experiments, we also examined the correlation between asymmetrical color filling-in and stimulus size of the colored semi-rings on the temporal, upper, and lower sides of the blind spot. To this extent, the width of one half ring was set at either 2.5° (same as in the main experiment), or 6.5° and 14°, in separate conditions, whereas the width of the half ring on the opposite side of the blind spot was kept constant at 2.5°. For greater accuracy, the panel for describing the spreading of the two colors consisted of five choices: 75, 60, 50, 40, and 25%. Since the asymmetrical color filling-in does not depend on the color combination (see Section Results), we only use red-green color pairs for this complementary experiment. Subjects were allowed enough time for any afterimages to disappear before entering a new trial and were asked to pay attention only to the colors inside the blind spot area.

### Data analysis

The eight potential choices in the bi-colored condition were categorized into five groups of category labels. A category label was defined as follows based on subject responses: “100%” stood for complete filling-in of the blind spot with either the nasal or upper color, corresponding to options III in Figures 1B,C. A label of “0%” defined complete filling-in from either the temporal or lower color with no contribution from the nasal or upper color, corresponding to option IV. “50%” was used when both colors of the annulus contributed equally to the filling-in as depicted by options I and II in the choice panel, regardless of the clarity of the border. “75%” represented a nasal- or upper-color dominance, corresponding to options V (75%) and VI (90%), while a label of “25%” represented either a temporal-or lower-color dominance, as reflected by options VII (10%) and VIII (25%).

For the statistical analysis, we computed the individual choice probability that a subject chose one particular category label. In the case of nasal versus temporal bipartite annuli (Figure 3), the probability for a subject to choose a nasal color dominance was calculated as follows: the number of times that the subject chose the category label of “75%” divided by 6 (the number of total configuration categories). To examine whether there was any statistical difference between different completion groups, we compared the choice probabilities across all 12 subjects using paired *t*-test analysis.

**Figure 2:**
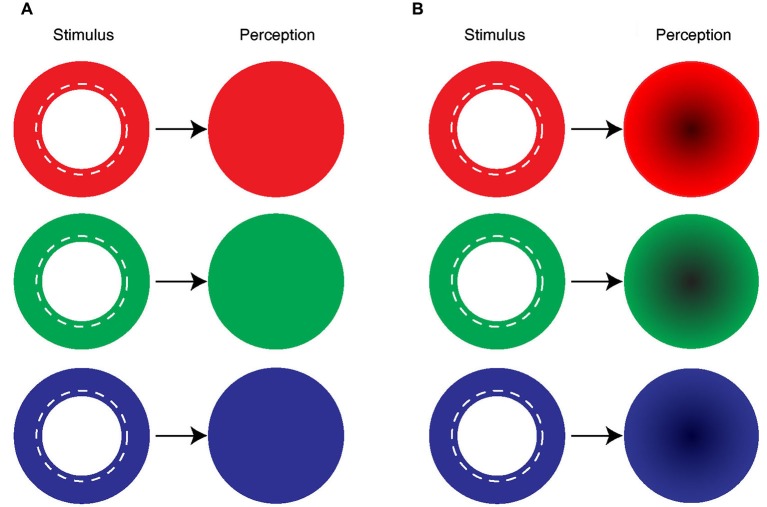
**Uniform and symmetrical filling-in by mono-colored ring. (A)** The majority of the subjects reported seeing a uniformly colored disk in their blind spot area when the blind spot boundary was covered by a mono-colored ring. **(B)** Some of the subjects described a uniformly colored disk with a darker center.

**Figure 3:**
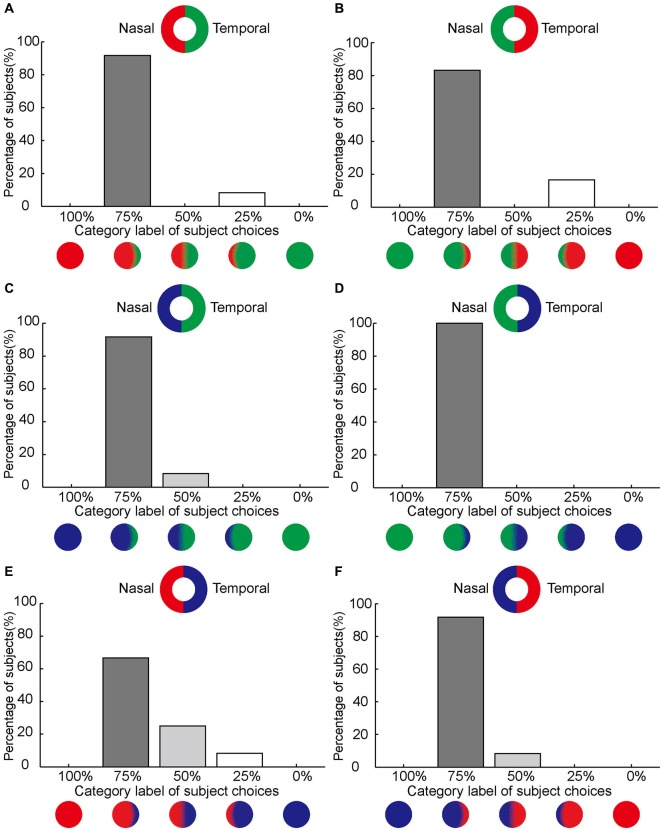
**Asymmetrical color filling-in of the blind spot.** **(A–F)** Demonstrate that color on the nasal side of the blind spot area dominates the filling-in with subjects typically choosing the 75% category label. The axis along the abscissas illustrates the category label of subject choices corresponding to the option panel shown in Figure 1 (see Section Materials and Methods). The axis along the ordinates shows the percentage of the subjects who chose a given option. Inset shows the stimulus. Notice that the position of the paired colors for red, green, and blue are reversed between **(A)**, **(C)** and **(E)** on one hand and **(B)**, **(D)**, and **(F)** on the other. Despite the change of sides, the nasal dominance of filling-in remains unchanged in the complimentary conditions.

In order to relate the nasal and the temporal half of the bi-colored annuli to their cortical representation, we calculated the cortical magnification for those two locations. We used the following empirical function from a previous study (Rovamo and Virsu, 1979):

(1) $$M\left(E\right)=7.99.\frac{1}{1+0.29E+0.000012E^{3}}$$

where *M* is the magnification factor at a particular eccentricity (*E*).

The blind spot was located in the temporal visual field between 13 and 21° with its center at 17° eccentricity across our 12 subjects. We defined a cortical magnification index (*C*_index_) for the two halves on the nasal and temporal side of the blind spot. The *C*_index_ for the half ring on the nasal side was simply computed as the ratio of the cortical magnification factors between the nasal half ring and the annuli (nasal plus temporal) along the horizontal meridian, using equation 2 as an example for annuli having a width of 2.5°:

(2) $$C_{index-nasal}=\frac{\left[M\left(10.5^{{}^{\circ}}\right)-M\left(13^{{}^{\circ}}\right)\right]}{\left[M\left(10.5^{{}^{\circ}}\right)-M\left(13^{{}^{\circ}}\right)\right]+\left[M\left(21^{{}^{\circ}}\right)-M\left(23.5^{{}^{\circ}}\right)\right]}$$

Similarly, the *C*_index_ for the temporal half ring with 2.5° width was computed by the following equation 3:

(3) $$C_{index-temporal}=\frac{\left[M\left(21^{{}^{\circ}}\right)-M\left(23.5^{{}^{\circ}}\right)\right]}{\left[M\left(10.5^{{}^{\circ}}\right)-M\left(13^{{}^{\circ}}\right)+M\left(21^{{}^{\circ}}\right)-M\left(23.5^{{}^{\circ}}\right)\right]}$$

This *C*_index_ is an indicator for the cortical projection of the stimulus in either nasal or temporal side of the blind spot.

## Results

We examined perceptual filling-in for mono- and bi-colored annuli surrounding the blind spot of the tested eye in 12 subjects. We found that the color from the nasal side predominantly filled in the blind spot, whereas the color from the temporal side filled in only a small portion. However, this asymmetry was absent when the bi-colored ring was rotated by 90°. Here, the two colors spread equally from the upper and lower sides of the blind spot and met approximately in the middle. These results suggest a retinotopic rule for the blind spot in that the strength of color filling-in decreases with distance from the fovea, thereby introducing a directional naso-temporal bias by virtue of the cortical magnification factor.

### Symmetrical filling-in of the blind spot using mono-colored annuli

All 12 subjects reported color filling-in with a mean response time of 1.31 ± 0.56 s (SEM) after the mono-colored annulus was presented around their blind spot. Specifically, subjects reported uniform and complete filling-in (Figure 2A). The numbers were: 7 out of 12 for blue, 11 for green, and 6 for red. This uniform color filling-in of the blind spot from the surround was consistent with earlier reports (Ramachandran, 1992a; Brown and Thurmond, 1993; Spillmann et al., 2006; Anstis, 2010). The subjects who did not see uniform filling-in (5 for blue, 1 for green, and 6 for red) reported that the center of the colored disk appeared darker than the periphery (Figure 2B). Nevertheless, all subjects reported symmetrical color filling-in of the blind spot with mono-colored stimuli.

### Asymmetrical color filling-in of the blind spot from nasal to temporal using bi-colored rings

For red/green bi-colored stimuli, 11 out of 12 subjects reported that the best match was the choice as the disk labeled with 75% category (Figure 3A). When the sides for red and green were between nasal and temporal (Figure 3B), the results were essentially the same (10 subjects out of 12). Furthermore, the asymmetrical color filling-in of the blind spot from nasal to temporal was found to be independent of the two colors paired: 11 out of 12 for blue-green, 12 for green-blue, 8 for red-blue, and 11 for blue-red (Figures 3C–F).

To further quantify this asymmetry, we combined the data from the six stimulus configurations (shown in Figure 3) for each subject and computed the choice probability for each category label to compare the mean choice probabilities for different choice labels in all 12 subjects. We found that subjects reported nasal color dominance significantly more often than either symmetrical filling-in (paired *t*-test, *P* << 0.001, *N* = 12) or temporal color dominance (paired *t*-test, *P* << 0.001, *N* = 12). This is shown in Figure 5A. These results demonstrate that the color of the bipartite annulus surrounding the blind spot on the nasal half spreads more extensively across the filled-in area than the color on the opposite (temporal) side.

**Figure 4:**
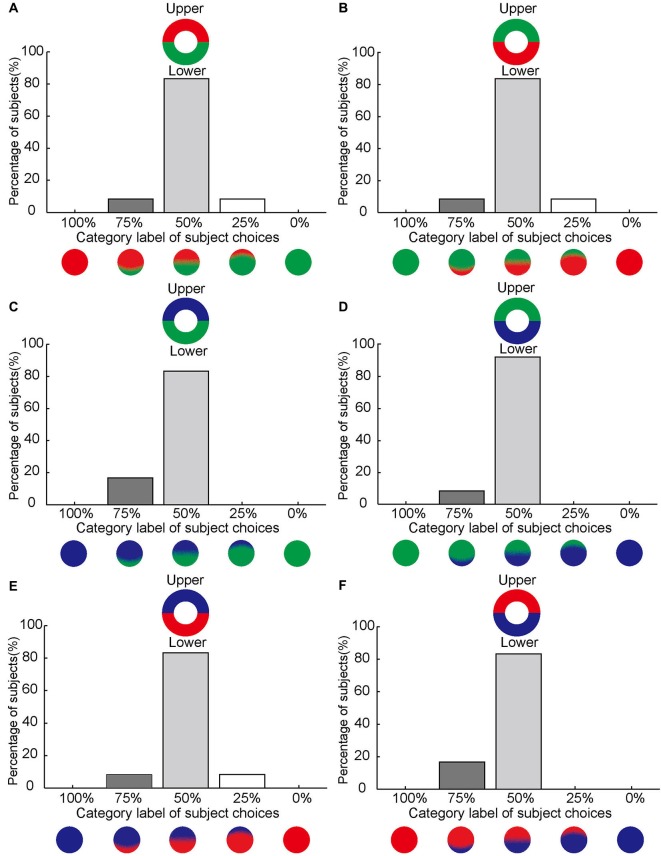
**No asymmetrical effect of color filling-in between upper and lower sides of the blind spot.** **(A–F)** Same format as Figure 3 but for stimuli subdivided into upper and lower halves.

**Figure 5:**
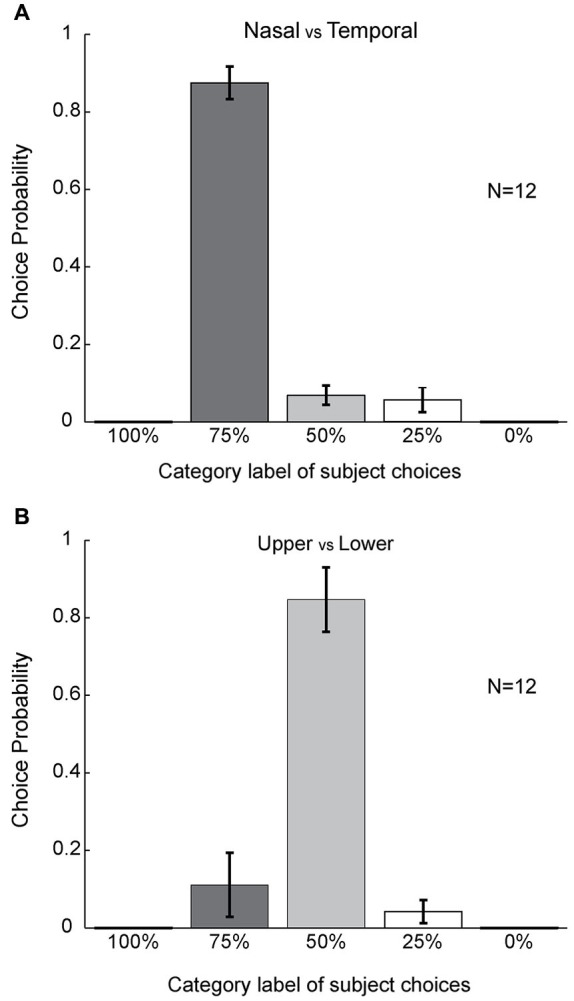
**Statistical analyses. (A)** Population level choice probability for the different choice categories. Choice probability here is defined as the percentage of choices for each category label (Figure 1) averaged across subjects (see Section Materials and Methods) (*n* = 12). Error bars refer to SEM values. Columns were compared using the paired *t*-test. **(B)** Population level of choice probability for upper versus lower cases. Statistics were performed using the same method as in **(A)**.

By contrast, asymmetrical color filling-in was absent when the bi-colored ring was rotated by 90° (Figure 1C). For this condition, most subjects reported equal filling-in by both colors, meeting approximately in the middle. This was true for all color conditions (10 out of 12 for red-green, 10 for green-red, 10 for blue-green, 11 for green-blue, 10 for blue-red, and 10 for red-blue; Figures 4A–F). When the data were collapsed across all conditions in the manner described above, we found with high statistical significance that subjects reported symmetrical filling-in more often than either upper color dominance (paired *t*-test, *P* << 0.001, *N* = 12) or lower color dominance (paired *t*-test, *P* << 0.001, *N* = 12). This is shown in Figure 5B. These results demonstrate that the colors of the upper and lower halves of the bipartite rings fill in symmetrically across the blind spot. Subjects did not report any “forbidden” (mixed) colors induced by the bi-colored rings.

### Asymmetrical color filling-in compensated and overruled by width of the colored half ring

**Figure 6:**
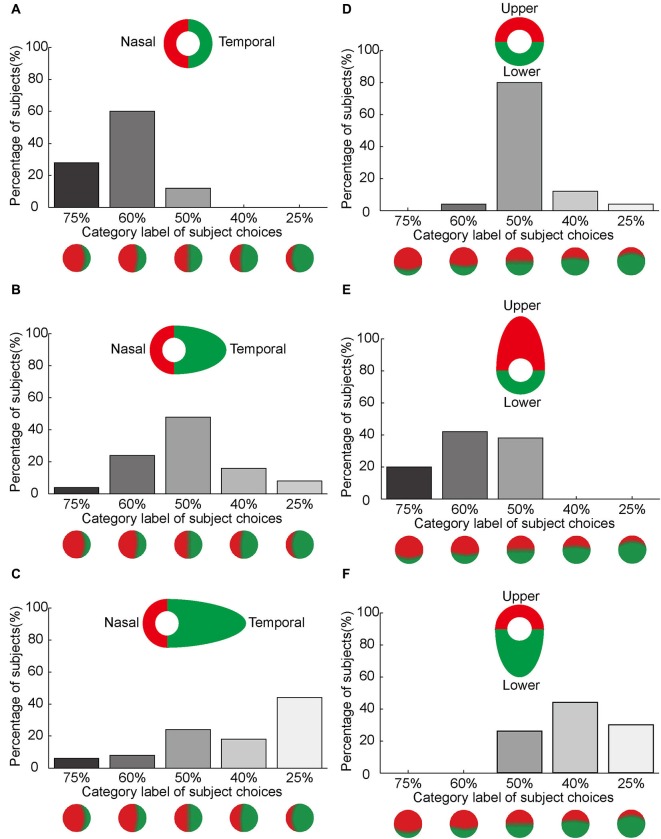
**Asymmetrical color filling-in by manipulation of the width of the colored half ring.** The axis along the abscissas illustrates the category label of subject choices. The axis along the ordinates shows the percentage of the subjects who chose a given label. Inset shows the stimulus. **(A–C)** illustrates the effect of increasing the annulus width on the temporal side of the blind spot on color filling-in. The width of the half ring on the nasal side of the blind spot was kept constant at 2.5°, whereas the width on the temporal side was set to 2.5° **(A)**, 6.5° **(B)** and 14° **(C)**, respectively. Notice that the nasal color dominance in **(A)** shifts towards equal dominance in **(B)** and temporal color dominance in **(C)** as the width of the temporal half ring increases. **(D–F)** illustrates the effect of increasing annulus width on the upper and lower sides of the blind spot. The width of the half ring was set to 2.5° for both sides **(D)**. A shift in color dominance occurs either from upper to lower **(E)** or from lower to upper **(F)** as the width increases.

Our subjects reported asymmetrical color filling-in of the blind spot from nasal to temporal using bi-colored rings, which may correspond to different cortical magnification factors associated with eccentricity. To test this hypothesis, we manipulated the size of the colored half rings around the blind spot. Figure 6 illustrates the primary observations in these experiments. Most of the subjects reported asymmetrical color filling-in of the blind spot from nasal to temporal when retested with bi-colored half rings of identical width (Figure 6A). This result was essentially the same as that in Figure 5A. Also as expected, most subjects reported equal color filling-in from the upper and lower sides of the blind spot (Figure 6D), consistent with earlier results (Figure 5B). However, when the width of the half ring on the temporal side of the blind spot was increased from 2.5° to 6.5°, as illustrated in Figure 6B, the previously observed asymmetrical color filling-in disappeared and was replaced with more or less equal filling-in from either side of the blind spot. When the size of the half ring on the temporal side was further increased to 14°, asymmetrical color filling-in reappeared, but now in the opposite direction (Figure 6C). More strikingly, when the size of the half ring in either upper or lower side of the blind spot was set to 6.5°, the majority of subjects reported asymmetrical color filling-in from the enlarged side (Figures 6E,F). These observations suggest that the difference in size of the cortical area receiving projections from colored halves with different widths plays an important role in the asymmetrical color filling-in of the blind spot. Moreover, a large difference in width can overrule the bias introduced by the difference in cortical magnification factor, suggesting that the size of the cortical projection from the stimuli surrounding the blind spot is an essential factor for determining the observed asymmetry of color filling-in.

### Asymmetrical cortical magnification factors for nasal and temporal sides of the blind spot

The *C*_index_ provides an estimate of the size of the cortical projection area corresponding to the proximal and distal sides of the annulus surrounding the blind spot. In the main experiments with equal halves of the annuli and a stimulus width of 2.5°, the *C*_index_ for the nasal half of the ring was 0.74, whereas the index for the temporal half was 0.26. These cortical indices were consistent with the observation that the nasal color dominates blind spot filling-in (Figures 3, 6A). In the complimentary experiments using unequal rings of 6.5°and 14° width on the temporal side of the blind spot, the *C*_index_ for the nasal half of the ring was 0.55 and 0.42, whereas the indices for the temporal half were 0.45 and 0.58, respectively. These indices were consistent with the change in color dominance from the nasal to the temporal side, when the width of the half ring on the temporal side of the blind spot was increased (Figures 6B,C). Altogether, these results suggest that the strength of filling-in decreases with distance from the fovea, and also correlates to the size of the surrounding stimuli of the blind spot that project to the visual cortex with different cortical magnification.

## Discussion

Our results show that color fills in the blind spot asymmetrically. The color bordering the nasal side of the blind spot spreads significantly farther than the color bordering the temporal side (Figures 3, 5A). The asymmetry of filling-in was independent of the two colors used and absent when the two colors bordered the upper and lower boundaries of the blind spot (Figures 4, 5B). In this case the upper and lower portions of the blind spot had the same distance from the fovea. These results suggest that the stronger filling-in from the nasal side observed for horizontally juxtaposed colors might be related to retinal eccentricity and the cortical magnification factor. The larger size could overrule greater eccentricity and smaller cortical magnification (Figure 6). A progressive decrease in sensitivity to color may be ruled out as an explanation as the color of the temporal half of the bipartite ring was perceived as vivid as that of the nasal half.

It is known that regions closer to the fovea are more extensively represented on the surface of the cortex than regions further away from it (Daniel and Whitteridge, 1961; Harvey and Dumoulin, 2011; Wandell and Winawer, 2011). In the experiments with unequal halves, subjects’ perception of the asymmetrical color filling-in could be canceled or even reversed, by a large increase in the width of the temporal half of the annulus (Figures 6A–C). Similarly, asymmetrical color filling-in of the blind spot could be induced by introducing a large asymmetry in size between the two halves on the upper and lower side of the blind spot where there was none before (Figures 6D–F). These observations suggest a retinotopic rule where the strength of color filling-in and hence the direction of filling-in of the blind spot is correlated to the size of the cortical projection area, which decreases with distance from the fovea. This was confirmed by the estimation of the cortical magnification (*C*_index_) on the nasal and temporal sides of the blind spot. Such a rule may also underlie a previously observed correlation between filling-in time or direction and size of an artificial scotoma (De Weerd et al., 1998; Shimojo et al., 2003; Kanai et al., 2006).

Compared with the filling-in of a natural scotoma such as the blind spot, the perceptual filling-in of an artificial scotoma takes longer to achieve. For example, Troxler type fading with strict fixation may take 10–15 s (Spillmann et al., 1984a; Ramachandran and Gregory, 1991; Pessoa and De Weerd, 2003; Komatsu, 2006). This is because fixational unsteadiness delays local adaptation of the target boundaries; thereby the time at which the surround signals can fill-in and render the stimulus invisible is prolonged. Accordingly, a two-stage model has been proposed to account for perceptual filling-in under these conditions: slow cancellation of the boundary followed by fast substitution by surround features (Spillmann and De Weerd, 2003; De Weerd, 2006). This active filling-in from the surround likely involves not only lateral propagation in the early visual system (Gilbert and Wiesel, 1992; Gilbert, 1992; Chino et al., 1995; Haynes et al., 2004; Meng et al., 2005), but also may recruits high-level neural mechanisms, possibly including modulation by attention (De Weerd et al., 1995, 2006; Mendola et al., 2006; Weil et al., 2012). By contrast, in a natural scotoma such as the blind spot, color filling-in is both rapid and preattentive. This is because the scotoma is fixed on the retina and there is little need to down-regulate the edge signal of the optic disk. Single cell recordings have revealed that neurons in layer 6 of area V1 not only respond to large stimuli that cover the blind spot, but also exhibit color selectivity (Komatsu et al., 2000, 2002).

Another natural scotoma exhibiting filling-in is the foveal blue scotoma (Magnussen et al., 2001, 2004). As there are no short-wave receptors in the foveola—the most inner part of the fovea—one should expect to see a dark spot, when we look at the blue sky. But this is not normally the case. Similar mechanisms of filling-in may underlie the two phenomena.

Instant filling-in of color is also observed for neon-color spreading (Redies and Spillmann, 1982; Grossberg and Mingolla, 1985; Bressan et al., 1997; Sasaki and Watanabe, 2004) and the watercolor effect (Pinna et al., 2001, 2003; Pinna and Reeves, 2006). In the latter, a faint color is induced on a large area by a chromatic double contour. The neuronal mechanism underlying watercolor spreading likely exists in early visual cortices. This is because most contour- or edge-detecting neurons are both orientation and color selective and the color signal is 5–6 times stronger for the edges than for the surface of the figure (Friedman et al., 2003; von der Heydt et al., 2003). Finally, a uniform spread of mono-color may also be responsible for color perception on extended surfaces (Kinoshita and Komatsu, 2001; Haynes et al., 2004; Huang and Paradiso, 2008).

In summary, by using bi-colored annuli surrounding the blind spot, our study suggests a retinotopic rule to account for the stronger filling-in of color from the nasal towards the temporal side of the blind spot, consistent with a difference in cortical representation. Future studies mapping the size of the activated brain areas during perceptual filling-in might be able to validate the hypothesis that the size of the cortical projection area is responsible for the observed asymmetry of perceptual filling-in.

## Author contributions

Hui Li, Junxiang Luo, and Yiliang Lu performed experiments; Lothar Spillmann and Wei Wang designed and supervised the study; Janis Kan, Lothar Spillmann, and Wei Wang wrote the manuscript.

## Conflict of interest statement

The authors declare that the research was conducted in the absence of any commercial or financial relationships that could be construed as a potential conflict of interest.
